# Vesicular traffic-mediated cell-to-cell signaling at the immune synapse in Ankylosing Spondylitis

**DOI:** 10.3389/fimmu.2022.1102405

**Published:** 2023-01-18

**Authors:** Fataneh Tavasolian, Chiara Pastrello, Zuhaib Ahmed, Igor Jurisica, Robert D. Inman

**Affiliations:** ^1^ Spondylitis Program, Division of Rheumatology, Schroeder Arthritis Institute, University Health Network, Toronto, ON, Canada; ^2^ Osteoarthritis Research Program, Division of Orthopedic Surgery, Schroeder Arthritis Institute, and Data Science Discovery Centre for Chronic Diseases, Krembil Research Institute, University Health Network, Toronto, ON, Canada; ^3^ Departments of Medical Biophysics and Computer Science, and the Faculty of Dentistry, University of Toronto, Toronto, ON, Canada; ^4^ Institute of Neuroimmunology, Slovak Academy of Sciences, Bratislava, Slovakia; ^5^ Krembil Research Institute, University Health Network, Toronto, ON, Canada; ^6^ Departments of Medicine and Immunology, University of Toronto, Toronto, ON, Canada

**Keywords:** ankylosing spondylitis, miRNA, immunological synapse, exosome secretion, vesicular trafficking

## Abstract

The chronic inflammatory disease ankylosing spondylitis (AS) is marked by back discomfort, spinal ankylosis, and extra-articular symptoms. In AS, inflammation is responsible for both pain and spinal ankylosis. However, the processes that sustain chronic inflammation remain unknown. Despite the years of research conducted to decipher the intricacy of AS, little progress has been made in identifying the signaling events that lead to the development of this disease. T cells, an immune cell type that initiates and regulates the body’s response to infection, have been established to substantially impact the development of AS. T lymphocytes are regarded as a crucial part of adaptive immunity for the control of the immune system. A highly coordinated interaction involving antigen-presenting cells (APCs) and T cells that regulate T cell activation constitutes an immunological synapse (IS). This first phase leads to the controlled trafficking of receptors and signaling mediators involved in folding endosomes to the cellular interface, which allows the transfer of information from T cells to APCs through IS formation. Discrimination of self and nonself antigen is somatically learned in adaptive immunity. In an autoimmune condition such as AS, there is a disturbance of self/nonself antigen discrimination; available findings imply that the IS plays a preeminent role in the adaptive immune response. In this paper, we provide insights into the genesis of AS by evaluating recent developments in the function of vesicular trafficking in IS formation and the targeted release of exosomes enriched microRNAs (miRNA) at the synaptic region in T cells.

## Introduction

**Figure 1:**
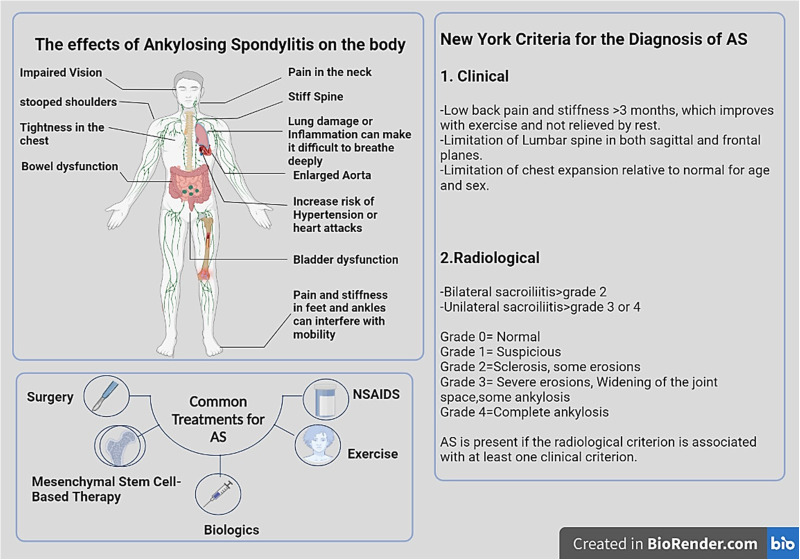
The physical manifestations of AS and a guide to diagnose and treatment. In AS, inflammation mostly affects axial joints, entheses, and extra-articular systems, including the uveal tract, digestive tract, mucocutaneous tissue, and heart. HLA-B27 is closely linked to spondyloarthropathies, particularly AS. In order to diagnose AS, at least one clinical indicator and one radiologic indicator are necessary. Bone scanning and magnetic resonance imaging (MRI) may help in the early detection of inflammation of the axial skeleton. Effective therapies for AS include local or systemic corticosteroid therapy, exercise, NSAIDs, DMARDs, methotrexate, azathioprine, anti-IL-17A monoclonal antibodies, TNF- antagonists, and MSCs therapy. Image was created with BioRender.com.

Spondyloarthritis (SpA) is a constellation of rheumatic disorders with comparable clinicopathological features (1). Ankylosing spondylitis (AS), a form of SpA, is an autoimmune disease involving the joints of the spine, the sacroiliac joints (SIJs), and the surrounding tendons and ligaments. In the absence of treatment, inflammation may lead to neo-ossification, which fuses the spine and restricts spinal movement (2, 3). Common clinical manifestations of AS include back discomfort, increased spinal stiffness, and inflammation of the hips, shoulders, and peripheral joints. Extra-articular manifestations (EAM) include acute anterior uveitis and inflammatory bowel disease (IBD). The objectives of treating AS are to alleviate symptoms, lessen functional limitations, maintain normal posture, and improve and maintain spinal flexibility. Nonsteroidal anti-inflammatory drugs (NSAIDs) and TNF inhibitors (TNFis) are the cornerstones of pharmaceutical therapy. Sulfasalazine, methotrexate, and non-TNFi biologics (secukinumab) are additional therapies. In addition, tofacitinib and filgotinib, two oral small-molecule JAK inhibitors, show promise in clinical trials and may soon be approved for AS (1, 4–7) (**Figure 1**).

## The role of HLA-B27 in AS

The development of AS is considered to occur from a complicated interaction between genetic predisposition and environmental circumstances (1, 8). Despite recent breakthroughs in understanding, the cause of AS remains poorly understood. According to research, other risk variables, including genetic background, immune response, microbiological triggers, and hormonal effects, have been linked to AS. Previously, genetic markers for prevalent susceptibility genes associated with immune regulation, including immunological synapse and T cell activation, have been studied. It is believed that AS is a hereditary disease, with HLA-B27 being the primary genetic risk factor (9). The Assessment of Spondyloarthritis International Society (ASAS) group’s recent suggestion of categorization criteria for patients without definite radiographic sacroiliitis is the result of updated data (10, 11). The criteria were based on two attributes: “imaging perspective,” patients with positive sacroiliitis on imaging (radiograph or MRI) and at least one SpA symptom, and “clinical characteristic,” HLA B27-positive individuals with at least two SpA symptoms (10–12).

Genome-wide association research has shown an abundance of additional loci (13), including ERAP1 and interleukin-23 receptor (IL-23R). HLA-B27 poses the highest risk and is identified in 90% of AS patients. However, only 5% of individuals with the HLA-B27 gene develop AS, suggesting that epigenetic mechanisms may be at play. AS may thus be a great example of a polygenic disease affected by epigenetics (14). Enhanced expression of HLA-B27 on APCs and CD4+ T cells is required for the development of AS, according to results from HLA-B27/β2 microglobulin transgenic (TG) rats (15). The conventional heterotrimeric MHC class I molecule consists of three non-covalently attached polypeptides: a highly polymorphic heavy chain (HC), a β2-microglobulin (β2m) light chain, and an 8- to 10-residue-long oligopeptide. In the absence of β2m, HCs may misfold and undergo endoplasmic reticulum (ER)-associated degradation. Misfolding and the production of dimers and multimers are common occurrences for HLA-B27. The three unique forms of dimeric MHC-I structures include cell surface HLA-B27 homodimers, intracellular MHC-I dimers, and exosomal MHC-I dimers. Exosomes are multivesicular bodies (MVBs) formed when endosomes proliferate inwards. A portion of these MVBs will fuse with the plasma membrane, therefore releasing intracellular vesicles into the extracellular environment. Different MHC-I dimers have been found on the surfaces of numerous cell types that produce exosomes (16, 17). It has been shown that the DCs of HLA-B27 TG rats have defective immunological synapse formation, which may be due to a number of physiological factors (18). Self-reactive T cells with poor immunological synapse formation may be able to circumvent negative selection in the thymus, where self-antigen expression is exceedingly low, owing to the extremely low expression of self-antigens. Numerous self- and foreign-antigen peptides have been analyzed and sequenced in the past, but there is no evidence that any of these peptides are cross-reactive (19). In addition, Taurog et al. observed that clinical symptoms manifested in HLA-B27/Hu2m transgenic rats lacking functional CD8+ T cells (20, 21). Creating a disease model for AS in which HLA-B27 exposes a peptide to CD8+ T cells is challenging (21, 22). In 2012, Glatigny et al. studied the molecular pathways underlying the diminished ability of dendritic cells (DCs) from HLA-B27 transgenic mice to form conjugates with naive T cells (23). They analyzed the interactions between CD4+ T cells and DCs generated by HLA-B27 transgenics (24). In the HLA-B27-transgenic rat model of spondyloarthropathy, researchers showed that mature HLA-B27 molecules expressed by DCs restrict the establishment of an antigen-independent immunologic synapse between naive CD4+ T cells and DCs by preventing the interaction of costimulatory molecules (24, 25). This process may impact the production and maintenance of Treg cells and contribute to the proliferation of pathogenic CD4+ T cells (26).

## Immunological synapse formation

By regulating leukocyte chemotaxis, migration, and T cell activation and by secreting exosomes to create the immune response, the IS contributes to a greater biological purpose (27). Within the immunological synapse, T cells interact with MHC-presented peptides on antigen-presenting cells, which are crucial biochemical interactions for regulating the immune response (28). If the IS is dysregulated and its connections are disturbed, aberrant immune activation may occur (29). Critical processes in immunological synapses are triggered by signaling in distinct micro-clusters of T cell antigen receptors and are essential for T cell development and effector functions. T cells commit to proliferating after interaction with APCs carrying antigens. Once committed, T cells proliferate quickly and, under the influence of lineage-specific cytokines, develop into several subsets of T helper cells (30–32). T cells establish several contacts of varying length and quality with APC throughout their scanning process. Some immunological synapses may only exist for a few minutes (33). When a T cell recognizes an APC containing homologous peptides, it stops its migration and forms an immunological synapse. During different phases of T cell activation, the actin cytoskeleton under the plasma membrane may link integrins, TCRs, and chemokine receptors, establishing a chemical and physical network that promotes cell-cell adhesion and enhances signal transmission (34). Cell adhesion molecules play a significant role in enabling these processes. Integrins are crucial modulators of immune cell activity during homeostasis and inflammation by mediating immune cell trafficking into tissues and developing immunological synapses (33). As part of the routine process of monitoring in lymph nodes and other organs, T cells form adhesions with APCs. The majority of these adhesions are caused by the binding of integrins to ligands on the surface of APCs. The degree of membrane contacts and intracellular signals that regulate the integrin’s conformation dictate the strength of integrin interactions.

T cells in the immunological synapse need prolonged stimulation to commit to proliferating, and they commit to proliferating after interacting with APCs carrying antigens. The cells that receive prolonged TCR activation inside a developing clone become polarized effectors, whereas the other cells remain nonpolarized. In response to specific cytokines, T cells proliferate rapidly and differentiate into several subsets of T helper cells. The cells travel to inflamed organs of the periphery, where they conduct effector activities and survive as a separate but smaller population of “effector memory” cells. Nonpolarized T cells that move to lymph nodes and react promptly to antigenic stimulation will proliferate after a brief TCR activation that is sufficient to induce commitment.

On the other hand, a persistent TCR activation in the presence of cytokines leads T cells to differentiate and move to inflamed peripheral tissues. Exosomes are secreted by immune cells and may govern the interplay between innate and adaptive immunity by influencing the link between them. Exosomes may be ingested by target cells, often by phagocytosis, and then fuse with their membranes to integrate membrane fragments into the host cell membrane and efficiently transport their cargo to the cytosol. Exosome-derived miRNAs are also recognized as crucial regulators of T-cell activity and differentiation. Extracellular signals, including co-stimulation and cytokine signals, dynamically control T-cell expression (35). As a consequence of these influences, the target cell’s phenotype and functioning may be altered. Exosomes are thus recognized as important intercellular communication intermediates, and the IS’s exosome-enriched miRNA release may also impact the immune response (36–38).

## AS is characterized by an altered immunological synapse

AS patients present high HLA-B27 expression levels (30). The processing and presentation of antigens occur sequentially. The multi-unit proteasome complex first degrades many proteins into peptide fragments of up to 25 amino acids, which are subsequently further degraded. TAP delivers antigen peptides of 8–16 residues into the ER (39). endoplasmic reticulum aminopeptidase 1/2 (ERAP1/ERAP2)/leucyl cystinyl aminopeptidase(LNPEP) will further cleave N-terminal extended precursors into oligopeptides of 8 or 9 residues, the ideal length for HLA-B27 binding (40). The peptides are then transported to the Golgi apparatus, where mature epitopes are produced. Other longer peptides may bind to HLA-B27, where they stay in the peptide groove with a protruding C-terminus or middle bulge. These peptides associated with HLA-B27 may be extremely immunogenic and evoke a T-cell response repertoire that is abnormally biased (41, 42).

The study on an animal model of AS has shown that DCs in HLA-B27 transgenic rats are dysfunctional (they lack class II MHC expression, leading to the elimination of a tolerogenic CD103+ population), which may promote the development of Th17 by inhibiting the creation of immunological synapses, hence aggravating the illness. CD4+T cells are discharged as naive CD4+ cells into the periphery, developing into Th1, Th2, Treg, and Th17 effector T cells. These effector T cells generate specific cytokines and transcriptional master regulators (30, 43, 44).

The IS induces T cell proliferation, expansion, and differentiation into cytotoxic and helper T cells. As indicated by establishing immunological synapses in AS, this process must be carefully managed and controlled (44). Most likely, the immunological synapse coordinates T-cell migration and activation, and the production of exosomes may affect the subsequent immune response. Exosomes transport several autoantigens linked with autoimmune disorders, including DNA and nucleosomes, DEK, α-enolase, citrullinated proteins, Sjögren syndrome-related antigen A (SSA), Sjögren syndrome-related antigen B (SSB) and Smith antigen (Sm) (45, 46). Exosomes secreted by stimulated or stressed cells or microbes have the potential to trigger autoimmunity. Exosomes may induce many inflammatory pathways, as supported by a large number of studies. However, uncertain is the degree to which exosomes have a role in initiating or sustaining the course of certain autoimmune disorders (47).

**Figure 2:**
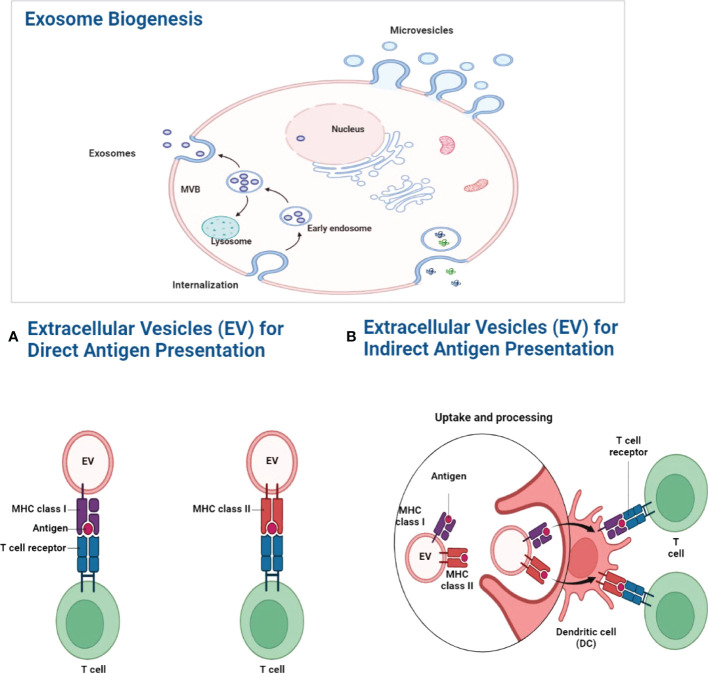
Exosome biogenesis. The MVB gradually fills with intraluminal vesicles as a consequence of inward budding. And these multivascular entities are capable of doing two tasks simultaneously. One of these two pathways may allow MVBs to combine with lysosomes and break down their contents. Alternatively, it may follow the exocytic route, fusing with the plasma membrane to discharge its contents into the extracellular environment. ILVs are referred to as “exosomes” after they are discharged into the extracellular environment. **(A)** Antigen presentation by exosomes. By expressing MHC molecules on their surface, exosomes may directly transport antigens to T cells. Exosome-associated peptide-MHC complexes are densely localized in the DC plasma membrane for efficient immunological synapses when exosomes bind to the surface of DCs. **(B)** Exogenous antigens are digested and presented onto MHC-I molecules by phagocytic antigen-presenting cells, which causes specific CD8+ T lymphocytes to cross-present MHC class I-restricted antigens (49).

Immune cells produce exosomes in the intracellular space for communication. These exosomes are generated inside the cell along the endocytic route by the inward budding of the endosomal membrane, which results in the formation of minute vesicular structures within the endosome lumen. Intraluminal vesicles (ILVs) may be exosomes that are pre-secreted and released into the extracellular environment following the fusion of so-called MVBs with the plasma membrane. Exosomes express MHC class I and II on their surface and are able to present antigens to T cells (48). Cross-dressing is a more efficient method of semi-direct antigen presentation that occurs when exosomes bind to the surface of DCs, where the DC plasma membrane concentrates a large number of peptide–MHC complexes for efficient immunological synapse formation. DCs endocyte exosomes, which lead to the intracellular processing and indirect presentation of antigens and peptides linked with exosomes, is another way of antigen presentation. Cross-presentation of MHC class I-restricted antigens to CD8+ T cells occurs when migratory DCs from the inflammatory environment migrate to the draining lymph nodes and, through synaptic vesicle transfer, convey antigens to conventional DCs in the lymph nodes (**Figure 2**) (49).

## Exosomes composition

The lipids, proteins, and RNAs in exosomes are different. Exosomal membrane lipids are abundant in sphingomyelin, cholesterol, glycosphingolipids, ceramide, phosphatidylserine, lysophosphatidylcholine, lyso-phosphatidylethanolamine, and phosphatidylcholine with short saturated fatty acids. The most abundant exosomal proteins include ESCRT machinery components (Alix, TSG101), heat-shock proteins (HSP 90/70), and tetraspanins (50). These proteins may largely contribute to the arrangement of receptors and other proteins inside exosomes. Due to their connection with lipid raft regions of the exosomal membrane, several proteins may also be packed into exosomes (51). In addition to carrying a variety of proteins, exosomes also transport cell-type-specific proteins. Exosomes may convey tumor antigens from tumor cells to dendritic cells (52). It has been proven that exosomes recovered from cells infected with various intracellular pathogens, including microbial particles, may enhance antigen presentation and macrophage activation (53).

Exosomes transport mRNA, miRNAs, and other short noncoding RNAs and long noncoding RNAs (54). Several studies have demonstrated that distinct miRNAs are expressed in innate and adaptive immune cells and play an essential role in the development and function of both types of immune cells, such as the regulation of inflammation and the modulation of T cells in AS (55). MiRNAs that target diverse signaling pathways regulate the differentiation of separate T-cell subgroups, resulting in differentiation start or inhibition/termination (56). The miRNAs operate as a group of gene regulators and may originate from either intracellularly modified expression or extracellular circulation. These circulating miRNAs may be transferred to IS *via* exosomes and transfer the signal to recipient cells, initiating an inflammatory signaling pathway in AS (57–60).

Sometimes, the RNA profile of exosomes is distinct from that of their parent cells, suggesting that the encapsulation of miRNA into exosomes is a competitive process (61). Multiple cell types create vesicles with similar miRNA contents, demonstrating the existence of a mechanism for selective miRNA export (62).

## Significance of exosomal miRNAs

Exosomal miRNAs may serve as diagnostic biomarkers, according to current studies. Even though exosome synthesis seems to be higher in tumors, exosomal miRNA transmission is also seen in healthy conditions, specifically between immune cells (63). Exosome-mediated transport of miRNAs from T cells to antigen-presenting cells controls gene expression in recipient cells at immunological synapses (64). Likewise, the transfer of miRNAs between mouse dendritic cells through exosomes was functional since they inhibited the translation of target mRNAs. It has also been shown that mRNAs, miRNAs, and cytokines delivered by exosomes produced from dendritic cells affect and interact with immune cells (65).

## What occurs subsequent to exosome packing?

The MVB migrates along microtubules and fuses with the plasma membrane at a cytoplasmic site during inward budding, releasing ILVs as exosomes into the extracellular environment (66). At least three mechanisms enable recipient cells to acquire exosomes from circulation. Initially, the exosomal membrane’s extracytoplasmic location may fuse with the plasma membrane of the receiving cell. By incorporating exosomal membrane and transmembrane proteins into the target cell’s plasma membrane, RNAs and proteins are released into the cytoplasm of the target cell (67). Second, endocytosis, which comprises clathrin-mediated endocytosis, caveolin-dependent endocytosis, lipid raft-mediated endocytosis, phagocytosis, and micropinocytosis, may integrate exosomes into the receiving cell. Following endocytosis, exosomes may fuse with the endosomal membrane or be transported to lysosomes for destruction (68). Third, upon adhesion to recipient cells, exosomes may remain stably attached to the plasma membrane, initiating signaling cascades by interacting exosome ligands with cell-surface receptors. Stable and extended cell surface exposure is probable, especially for cells with little or no endocytic activity (69, 70).

## The function of exosomal miRNA in the IS

It has been shown that the IS functions as a channel for cell-to-cell interactions involving vesicular traffic and an active location for releasing soluble chemicals. The vesicular trafficking component may be engaged in synapse construction and the targeted release of microvesicles, which function as synaptic transmission facilitators (71). The immunological synapse is also the releasing location for canonical, MVB-derived CD63+ exosomes rich in miRNAs. As discussed before, signaling molecules, cytoskeletal components, and organelles must be rearranged in a temporally and spatially specified way in order for the IS to form (72). The IS membrane is arranged in concentric domains known as supramolecular activation clusters (SMACs), with the central supramolecular activation cluster (cSMAC) enclosing the TCR and associated proteins (TCR signalosomes) and the peripheral and distal supramolecular activation clusters (pSMAC and dSMAC, respectively) (73, 74). The pSMAC is rich in integrins, such as LFA-1, and cytoskeleton-binding proteins, such as talin, while the dSMAC is rich in F-actin and proteins with large ectodomains, such as CD45 and CD43 (75, 76). Exosome-like extracellular vesicle compartments (exo-cSMAC) have recently been found in synaptic regions rich in miRNA, DNA, and proteins. Exosomes bearing TCR are picked up by their respective APCs. They perform out signaling and allow intercommunication among T cells and APCs by releasing miRNA-loaded exosomes from T cells, which control gene expression in APCs (77, 78).

**Figure 3:**
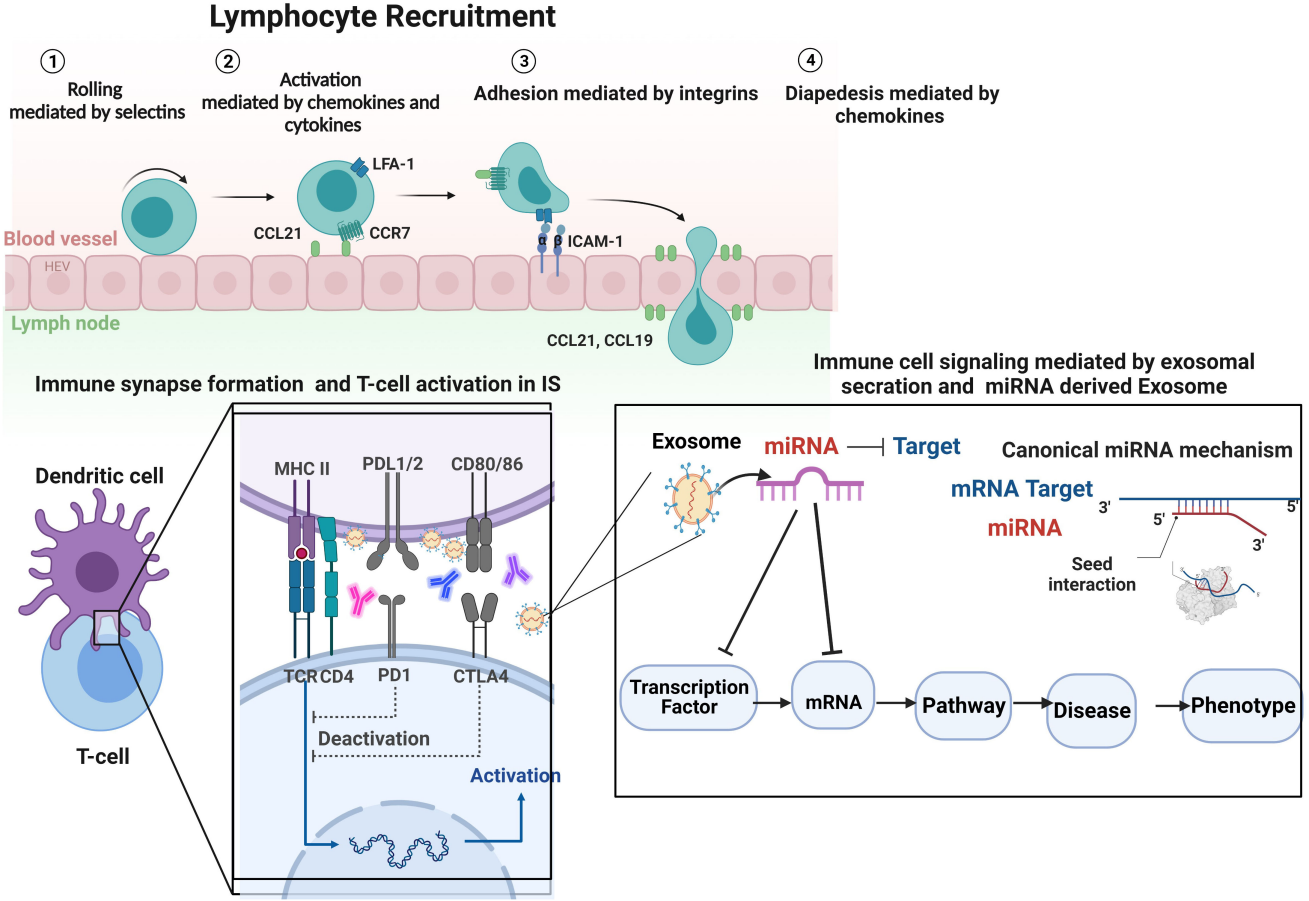
Regarding immune cell activity, integrin participation may be divided into three types of processes: immune cell recruitment, immune cell interactions, and immune cell signaling, followed by the formation of IS and biogenesis and secretion of exosomes in IS. T cell function is affected by three distinct activation signals: (1) recognition and binding of the antigen peptide (Ag) by the T cell receptor (TCR), resulting in the formation of the APC-T cell complex; (2) adjustment of the synapse by proteins CD28 and CD80/CD86; and (3) molecular interaction signaling APC release of inflammatory cytokines and exosomes, which activate T cell differentiation. A platform hypothesizes the potential impact of immune cell-derived exosomes containing miRNA on immune response initiation and immune system function and reprogramming the cells, which explains the model that prioritizes diseases and phenotypes based on miRNA-mRNA-disease associations. By analyzing the features of exosomes in individuals with AS in terms of the possibilities of utilizing exosomes as biomarkers and finding potential liquid biopsies, it may be possible to acquire a better understanding of the pathophysiology of AS. Future research will concentrate on miRNA-containing exosomes and their interactions with T cells to help address this essential knowledge gap. Image was created with BioRender.com.

Notably, exosomes in the IS may contribute to immune response induction. T cell activation is governed by the interaction between T cells and dendritic cells (DCs), which begins the immunological response (79). T cell activation needs antigen presentation on immune cell surfaces, the formation of a synapse, and the particular detection of a complex containing three activating signals by T cells. Signal 1 is the antigenic stimulation conveyed by MHC molecules carrying peptides; in this case, exosomes can involve expressing MHC molecules on their surface taking antigenic peptide (**Figure 3**), while signal two is sent by costimulatory molecules, which may include exosomes since they also express costimulatory molecules. Signal 3 originates from cytokines generated by DCs or other sources, including exosomes and their cargo of proteins and miRNAs (**Figure 3**) (49, 75, 80, 81). In an autoimmune disease that involves dysregulation of self/non-self-discriminating, the importance of the immunological synapse would be expected. In addition, this model provides a framework for hypothesizing the potential role of miRNA-exosome in the beginning and modulation of the immune response in the immunological synapse of AS (30). Multiple cell-surface-expressed receptors and markers may be incorporated into the outer layer of exosomes and delivered to the target cell membrane through membrane fusion. This approach can potentially affect the phenotype and many biological characteristics of the transplanted recipient cells. Consequently, cells communicate by delivering receptors to target cells or moving cargo consisting of mRNA, miRNA, proteins, or other macromolecules from one cell to another (73, 82) (**Figure 3**).

## Viewpoints on the current knowledge and future perspectives of the immune synapse in AS

The essential function of the HLA molecule is inducing and controlling immune responses. About 30% of the heritability of AS is attributed to HLA-B27 (83). It is necessary to determine the synapse’s signaling function in AS (30). Integration of experimental studies and computational models is required to elucidate the interplay of complex competing effects in different aspects of T-cell signaling. Computational models can explore and consider outcomes of various mechanistic hypotheses for each signaling component and determine whether or not individual hypotheses produce results consistent with experimental observations (84). Such analysis must be sufficiently powered, and independent data sets are required for validation before biological validation. *In vitro*, functional studies can provide final validation of these models and an in-depth analysis of different subtypes of AS. We considered available datasets from the Gene Expression Omnibus (GEO) database (http://www.ncbi.nlm.nih.gov/geo) for “ankylosing spondylitis”, included only series analyzed in *Homo sapiens* and *Mus musculus* and through expression profiling by array or sequencing. Only a few datasets related to AS in GEO met these criteria as of October 2022. Blood samples, synovial biopsies, and mesenchymal stem cells are all included in these datasets, but exosomes are not. Notably, during immunological cell-cell contacts, exosomes transfer to exchange chemicals. Exosome research has the potential to reveal unidentified cellular and molecular pathways of intercellular communication in AS. Although the precise process by which exosomes and their cargo are absorbed by a recipient cell is unknown, the mechanism and subsequent destiny of the cargo seem to be cell-type and environment-dependent. We hypothesize that the immunological synapse and other forms of intercellular connections facilitate the precise transfer of exosomes to guarantee the effective delivery of their cargo, notably genetic material in the form of miRNAs. Since exosomes are potential delivery systems for gene therapy in immune system disorders like AS (85–90), a thorough knowledge of how exosomes are transferred between immune cells would enable their therapeutic exploitation. Given that exosomes would be released as extracellular vesicles, they may represent an important intercellular communication method. Therefore, the exosomal fully-folded MHC I dimers may transmit signals to the resident cells in entheses to induce inflammation, which may lead to alterations in the joint architecture and the formation of new bone.

## Conclusion

Variations in vesicular flow are critical elements in T cell-mediated diseases. Current knowledge of IS assembly mechanics supports the notion of the IS as being a promising pharmacological target. Drugs that target the activities of molecules involved in IS production and alter the immune response in AS hold promise for future AS treatments. This review paper focused on evolving concepts of IS formation and mediators secreting in IS, like exosomes and their cargoes, including miRNAs. We emphasized the epigenetically significant role of exosomal miRNA modulation of the immune response. Future research may investigate the epigenetic effects on the pathophysiology of AS and how exosomal miRNAs may alter gene expression in recipient cells.

## Author contributions

All authors conceived of the presented idea and developed the theory. All authors contributed to the article and approved the submitted version. This review paper is directly supervised by RI.
